# Raised HIF1α during normoxia in high altitude pulmonary edema susceptible non-mountaineers

**DOI:** 10.1038/srep26468

**Published:** 2016-05-23

**Authors:** Poonam Soree, Rajinder K. Gupta, Krishan Singh, Koundinya Desiraju, Anurag Agrawal, Praveen Vats, Abhishek Bharadwaj, T. P. Baburaj, Pooja Chaudhary, Vijay K. Singh, Saroj Verma, Amir Chand Bajaj, Shashi Bala Singh

**Affiliations:** 1 Defence Institute of Physiology and Allied Sciences, Timarpur, Delhi 110054, India; 2 CSIR Institute of Genomics and Integrative Biology, Mall Road, Delhi 110007, India

## Abstract

High altitude pulmonary edema (HAPE) susceptibility is associated with EGLN1 polymorphisms, we hypothesized that HAPE-susceptible (HAPE-S, had HAPE episode in past) subjects may exhibit abnormal HIF1α levels in normoxic conditions. We measured HIF1α levels in HAPE-S and HAPE resistant (HAPE-R, no HAPE episode) individuals with similar pulmonary functions. Hemodynamic responses were also measured before and after normobaric hypoxia (Fi02 = 0.12 for 30 min duration at sea level) in both groups. . HIF1α was higher in HAPE-S (320.3 ± 267.5 vs 58.75 ± 33.88 pg/ml, P < 0.05) than HAPE-R, at baseline, despite no significant difference in baseline oxygen saturations (97.7 ± 1.7% and 98.8 ± 0.7). As expected, HAPE-S showed an exaggerated increase in pulmonary artery pressure (27.9 ± 6 vs 19.3 ± 3.7 mm Hg, P < 0.05) and a fall in peripheral oxygen saturation (66.9 ± 11.7 vs 78.7 ± 3.8%, P < 0.05), when exposed to hypoxia. HIF1α levels at baseline could accurately classify members of the two groups (AUC = 0.87). In a subset of the groups where hemoglobin fractions were additionally measured to understand the cause of elevated hypoxic response at baseline, two of four HAPE-S subjects showed reduced HbA. In conclusion, HIF 1 α levels during normoxia may represent an important marker for determination of HAPE susceptibility.

Pulmonary vasculature constricts in response to hypoxia at high altitude. It is well known that HAPE-S (past HAPE episode) demonstrates an exaggerated pulmonary vascular response (PVR) on exposure to acute hypoxia^1^,^2^,^3^. Genetic variations in EGLN1 (HIF-prolyl hydroxylase 2), which normally hydroxylates HIF1α and marks it for degradation during normoxia, have been associated with HAPE^4^. HIF1α is known to cause vascular remodeling due to chronic hypoxia. Chronic hypoxia exposure sensitizes pulmonary vasculature for exaggerated pressor response to acute hypoxia exposure in animals and humans^5^,^6^,^7^. HAPE-S subjects when given acute hyperoxia at high altitude failed to show any reduction in pulmonary hypertension (PH). This suggested existence of structural changes in pulmonary vasculature due to vascular remodeling in HAPE-S subjects^8^,^9^. All these studies suggested that chronic hypoxia exposure is essential for development of exaggerated PVR to acute hypoxia thus may contribute to hypoxia intolerance.

We therefore hypothesized that some constitutional abnormality resulting in chronically high levels of HIF 1 α exists in individuals who show exaggerated PVR to acute hypoxia. We measured HIF 1α level, basal P50, and T_3_ in HAPE-S and compared with HAPE-R subjects. Elevated HIF 1α level, right shift in P50, or rise in T_3_ are potential indicators of tissue hypoxia resulting in increased hypoxia response^10^,^11^,^12^. Abnormal hemoglobin fractions may reduce oxygen transport, resulting in chronic hypoxia. Therefore, they were also measured as a secondary goal in a subset group.

## Result

### Baseline anthropometry, duration of high altitude stay and pulmonary function

Baseline anthropometry, duration of high altitude stay and pulmonary function are summarized in Table 1. There were no significant differences in age, height and weight, between two groups. There were no significant differences in forced vital capacity (FVC), Forced expiratory volume in 1 sec (FEV1), FEV1/FVC, Total lung capacity (TLC), Functional residual capacity (FRC), pulmonary diffusion capacity for carbon monoxide (DLCO) and Alveolar ventilation (VA), DLCO/VA between two groups.

### Hemodynamics before and after hypoxia

HR and Ppa (Formula No. 1) was high in HAPE-S during normoxia summarized in Table 2. Hypoxia led to an increase in HR and Ppa and a fall in Spo_2_ in both HAPE-S and HAPE-R. However HAPE-S showed an exaggerated rise in HR, Ppa and a fall in Spo_2_ compared to HAPE-R, as expected.

### Baseline venous blood gas and biochemical parameters

**Figure 1:**
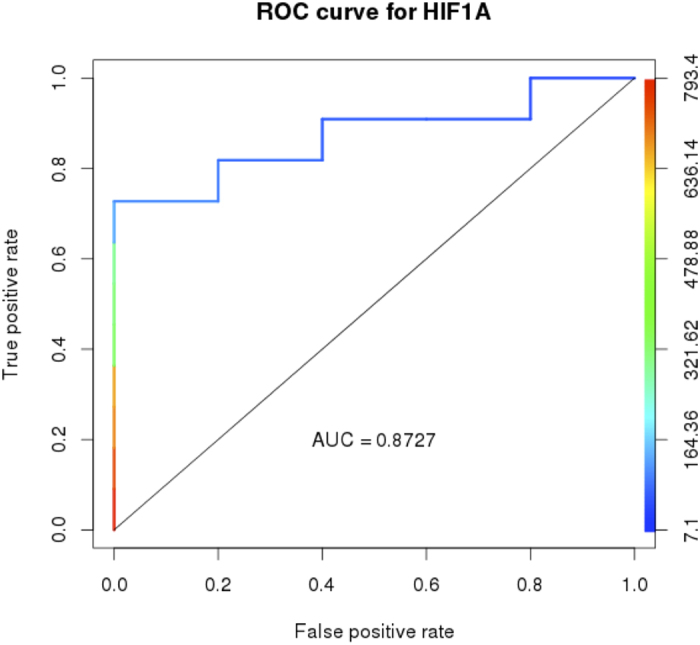
Receiver operator curve indicating the AUC for baseline hypoxia inducible factor (HIF1α) levels. Curves are color coded based on cutoff values which are shown as the second Y-axis.

To determine whether there was chronic elevation of the hypoxic response in HAPE-S subjects, we measured HIF 1α in venous blood. HAPE-S subject showed high (p < 0.05) baseline levels of HIF 1α (320.3 ± 267.5 vs 58.75 ± 33.88 pg/ml) compared to HAPE-R, suggesting elevated hypoxic response. The receiver operator characteristics of serum HIF 1α level as a discriminator between HAPE-S and HAPE-R were excellent (Fig. 1, AUC = 0.87).

To determine whether there was any evidence of downstream adaptive changes to a chronic hypoxic response, we measured venous blood oxygen and pH, oxygen affinity of hemoglobin (P50: Formula No. 2), 2–3 DPG, thyroxine and thyroid stimulating hormone (T_3_, TSH). Venous blood oxygen was higher in HAPE-S (HAPE-S vs HAPE-R; Pvo_2_, 24.91 ± 5.45 vs 16.09 ± 4.21 mm Hg; Svo_2_, 36.1 ± 13.48 vs 19 ± 7.89%). Although direct downstream markers of tissue hypoxic response were variable (normal pH and 2–3 DPG), but a right shift in the oxygen-binding curve of hemoglobin (P50, 26.38 ± 1.58 vs 24.4 ± 3.16 mm Hg) and elevated T_3_ (1.35 ± 0.29 vs 1.01 ± 0.35 pg/dl) levels in HAPE-S supported tissue hypoxia. There was also some evidence of cardiovascular strain with elevated atrial natriuretic peptide (ANP). Table 3 summarizes the venous blood gas and biochemical data. Figure 1 and Table 3.

Abnormal hemoglobins were seen in two of four HAPE-S subjects, where blood was available for hemoglobin profiling. Table 4 summarizes HbA and HbA_2_ levels of this subset. When compared to expected frequencies of hemoglobinopathies in healthy Indians (less than 10%), this proportion (10%) is marginally significant (p = 0.05, one tailed distribution) but is preliminary in nature due to extremely small sample size.

## Discussion

To the best of our knowledge we are the first to show the likelihood of a chronically upregulated hypoxic response resulting in development of subclinical pulmonary hypertension (Ppa < 20 mmHg) in HAPE-S^13^. Previous studies on susceptibility to HAPE were done on mountaineers and experimental protocol involved rapid ascent to high altitude. It is well known that physical exertion associated with rapid ascent is an independent factor for occurrence of HAPE and this along with selection bias may account for negative findings regarding baseline differences in pulmonary vascular function^14^,^15^. In contrast, present study on Indian armed forces personnel are routinely deployed at high altitude location in the Himalaya mountains, without any previous mountaineering experience, but following a carefully controlled acclimatization schedule that lowers the risk of HAPE. Therefore, it is difficult to conduct prospective study on HAPE susceptibility in Army personnel.

A few limitations of our study merit further discussion. First, subjects were studied after the development of HAPE since prospective studies are not feasible as discussed above. This raises a question of whether the elevated hypoxic response is a consequence of having suffered from HAPE. We consider this unlikely because it is well known that HAPE is a reversible noninflammatory edema which quickly resolves with descent^16^. There was also no persistent pulmonary membrane defect in HAPE-S since DLCO/VA was not significantly different between two groups in accordance with a previous study^17^. Further, all the subjects were staying at sea level preceding six months of baseline data collection, ruling out persisting hypoxic stress. Another limitation is that the sample size of the present study is too small for understanding the etiology of the elevated hypoxic response, with hemoglobin fraction profile being done in only four HAPE-S subjects. This was dictated by the practical feasibility of testing for a number of parameters in the limited amount of samples available and the fact that testing for abnormal hemoglobins was not our initial priority. The study also has many positives. This is the first such study, to our knowledge, in a cohort of HAPE-S subjects from a non-mountaineer population who followed acclimatization schedule on arrival at high altitude. This increases the likelihood that HAPE-S subjects from this population are representative of a true HAPE susceptible population with underlying abnormalities that predispose to exaggerated responses to hypoxia, even if the precise cause remains undefined. Also, while our hemoglobin data is not conclusive, it is certainly interesting that half of the subjects tested in the HAPE-S group (2 of 4) had clearly abnormal hemoglobin profiles, something that is statistically unlikely (less than 5% probability) in a random sample from a population where the background prevalence is less than 10%.

In our small study, baseline elevation of HIF1α in blood had good receiver operating characteristics (AUC 0.87) as a marker of HAPE-risk, 80% sensitive and 81.45% specific using a threshold of 86.45. The precise source of HIF1α **is** not clear but there is substantial evidence that cells with elevated hypoxic response secrete HIF1α extracellularly via microvesicles/exosomes as part of cell-cell signaling^18^. We did not find any significant differences in lung function or baseline arterial oxygen saturation that would explain the elevated hypoxic response. However, elevation of hypoxic response would reduce mitochondrial oxygen utilization and presumably increase venous saturation, if arterial oxygen delivery were the same. This was observed in our baseline data (Table 3) supporting a functional upregulated hypoxic response in accordance with previous study^19^. We did observe a right shift in P50, without any rise in 2–3 DPG concentration, in HAPE-S subjects^11^. Shappell and co-workers reported that reduced oxygen affinity (right shift in p50) of hemoglobin occurs within a minutes in coronary sinus blood during atrial pacing in patients with angina pectoris without measurable change in the factors known to influence p50^20^. The relationship between fall in PaO_2_ and rise in 2–3 DPG become evident only when Pao_2_ becomes less than 60 mmHg and a right shift in p50 may be a more sensitive index of hypoxia^21^,^22^. Previous studies have shown that hemoglobin with lower oxygen affinity is related to poor hypoxia tolerance at high altitude probably due to reduced oxygen delivery to tissues^23^,^24^. Also studies suggested that mutation in hemoglobin causing increase in oxygen affinity increases tolerance to hypoxia^25^,^26^. A lower HbA fraction in HAPE-S as seen for subjects 1 and 2 (Table 4) can cause hypoxia intolerance. Studies showed that Hb E heterozygous individuals have reduced exercise tolerance in accordance with HAPE-S^27^ and β Thalassemia patients have elevated HIF1α and GLUT1 levels^28^. However, as discussed above, it cannot be concluded from only two of four HAPE-S subjects that elevated HIF1α is due to the less HbA fraction. While it remains possible that genetic variation in the hypoxia response pathway may account for elevated HIF1α levels in HAPE-S, such studies could not be performed in this group at this time. Both these hypotheses need to be tested further.

The differences in biochemistry profile seen between HAPE-S and HAPE-R are mostly in accordance with molecular programs known to be associated with the cellular hypoxic response. We observed high levels of T3, without changes in TSH levels, in HAPE-S subjects, which is consistent with known changes to the thyroid profile in hypoxemic COPD patients^10^. At a molecular level, T_3_ stimulates HIF-1α to initiate transcriptional programs involved in angiogenesis, vascular remodeling, erythropoiesis, vasomotor reactivity and vascular tone^29^,^30^,^31^. Together our findings are most consistent with a model where chronic hypoxic response induced vascular remodeling may be responsible for baseline high Ppa and exaggerated hypoxic pulmonary vasoconstriction (HPV) in subjects at increased risk of HAPE. Numerous investigations of human and animal model have shown that chronic hypoxia is a trigger of pulmonary vascular remodeling^9^. Pulmonary hypertension also coexists in various hemoglobinopathy disorders associated with hypoxemia^32^. Elevated ANP levels could also be explained by sub clinical pulmonary hypertension type changes that we have recently described in HAPE-S subjects^33^,^34^,^35^,^36^. It is important to reiterate here that the chronic hypoxic response being discussed here is most likely disassociated from any severe systemic blood or tissue hypoxia. Neither do we see any arterial or venous reduction in oxygen saturation, nor is it likely that highly fit army soldiers suffer from true hypoxia. Based on a recent study where 10% Caucasians showed exaggerated HPV and 13% of them suffered HAPE when rapidly inducted to 4559 m^37^. We speculate that HAPE susceptibility represents molecular dysregulation of a physiological stress response pathway that may be triggered by factors including but not limited to abnormal hemoglobins.

In summary, baseline elevation of HIF1α is associated with HAPE susceptibility. The potential use of HIF1α as a screening marker for HAPE susceptibility is attractive because of ease of testing but mechanistic understanding is needed.

## Methods

The average duration of high altitude stay of HAPE-S participants was 6 month to one year. The altitude of previous onset of HAPE in susceptible subjects was 3500 m and diagnosed radiographically. HAPE-R are individuals who do not suffered HAPE during their high altitude tenure of 2 year which included 3 month stay at extreme altitude >4500 m. The participants of both the groups were air inducted to 3500 m from sea level. All the participants of both the groups followed acclimatization schedule for the initial six days of induction to high altitude which involve complete rest for first two days followed by graded increase in physical activity. All participants were healthy, nonsmokers, lowlanders and receiving no medication at the time of study. None of the participants has resided above 2000 m within last six months before baseline measurements were carried out in Delhi, India at an altitude 293 m above sea level.

Anthropometry, pulmonary functions and hemodynamic parameters were measured in HAPE-S and HAPE-R (n = 11 each group) to investigate whether HIF1α levels can be related with HAPE susceptibility. Due to limited amount of blood available and technical requirements of some tests, not every test could be performed for every HAPE-R subject but n was greater or equal to five in each group. Therefore, venous blood gas and biochemical parameters were measured in HAPE-S (n = 11) with variable number of HAPE-R subjects for different parameters (n = 11 for pH, Pvo_2_, Svo_2_, P50; n = 7 for T3, T4, TSH and 2–3 DPG ; n = 9 for ANP; n = 5 for HIF1α). In a subset of HAPE-S (n = 4) hemoglobin fractions were measured to understand the cause of elevated hypoxia response.

All experimental protocols were approved by Defence Institute of Physiology and Allied Sciences Ethics Committee for scientific experiments. Informed written consent was obtained from all participants before enrollment in the study. All methods were carried out in accordance with the approved guidelines and regulations.

### General procedure

The subjects were investigated in supine position for hemodynamic studies while breathing synthetic gas mixture consisting of 21% or 12% oxygen (Fio2 = 0.12) mixed in nitrogen by use of hypoxicator system (GO2 altitude®, Biomedtech Australia). Inhalation was performed via a tight fit face mask. Heart rate (HR), Systolic blood pressure (SBP), diastolic blood pressure (DBP), peripheral oxygen saturation (Spo_2_) were recorded before and end of 30 minute of hypoxia stress by using Multipara meter monitor, BPL, India.

### Pulmonary function measurements

Spirometery and single breath diffusion capacity was performed using Easy one Pro system (Ndd Medizintechnik AG CH-8005 Zurich, Switzerland, Germany).

### Determination of pulmonary artery systolic pressure (sPpa)

sPpa can be reliably measured by echocardiography^38^.Two dimensional and Doppler echocardiography recordings were obtained with conventional equipment (My Lab 30 Gold line ultrasonogarph, Esaote India). Flow velocities of tricuspid valve regurgitation jets were measured at the highest coherent boundary of the spectral wave using continuous-wave doppler, guided by colour-flow doppler for sPpa determination. Measurements were recorded during normoxia at rest and after 30 min of hypoxia exposure.

Pulmonary artery systolic pressure (sPpa) was calculated as follows


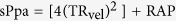


Where TR_vel_ is tricuspid regurgitation jet velocity and RAP is the estimated right atrial pressure based on respiration variation in inferior vena cava size.

Calculation of Mean pulmonary artery pressure


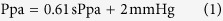


Measurements of HR, SBP, DBP, Spo_2_ and sPpa were obtained before and at the end of 30 min. of hypoxic stress.

### Hematological and biochemical studies

Baseline venous blood sample was collected in heparin tube.

### Blood gas analysis for calculation of p50

Immediately two drops of blood were transferred to i-STAT cartridge EG7 + (blood gas analyzer i-STAT Abbott, USA)^39^ for estimation of venous blood gas parameter.

Calculation of P50


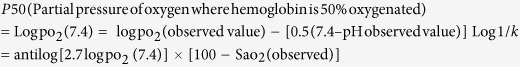



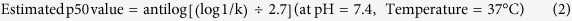


Where pH, Po_2_ (partial pressure of oxygen), Sao_2_ (saturation of oxygen) in venous blood^40^,^41^,^42^.

### Estimation of T_3_, T_4_, TSH, ANP, HIF1α and 2–3 DPG levels

3 ml of whole blood was centrifuged at 3000 rpm for 30 minutes in heparin vaccutainer. After centrifugation, supernatant plasma was used for biochemical estimation by using Enzyme Link Immuno-Sorbant Assay based kits.

Plasma total triiodothyronine concentration (T_3_, ng/ml) was measured by enzyme-immuno assay method (DSI S.r.l; TH-152). The sensitivity of assay was 0.2 ng/ml.

Plasma total Thyroxine concentration (T_4_, nmol/l) was measured by enzyme-immuno assay method (DSI S.r.l; TH-252). The sensitivity of assay was 5 nmol/l.

Plasma Thyrotropin concentration (TSH, μIU/ml) was measured by enzyme-immuno assay method (DSI S.r.l; TH-351). The sensitivity of assay was 0.05 μIU/ml.

Plasma ANP levels (pg/ml) was measured by enzyme-immuno assay method (Elabscience; E-EL-H0532). The sensitivity of assay was 4.68 pg/ml. The coefficient of variation were < 10%.

Human hypoxia inducible factor 1α concentration in serum (HIF-1 α, pg/ml) was measured by enzyme-immuno assay method (CUSABIO; CSB-E08539h). The sensitivity of assay was 7.81 pg/ml. CV < 10%.

Plasma 2–3 DPG levels (nmol/ml) was measured by enzyme-immuno assay method (Wuhan Newqidi biological technology co Ltd). The sensitivity of assay was 0.01 nmol/ml. The coefficient of variation were < 8%.

### Quantification of Hemoglobin fraction using high performance liquid chromatography (HPLC)

3 ml of whole blood for quantification of hemoglobin fraction by high performance liquid chromatography (HPLC, Bio-Rad D10) system^43^,^44^,^45^.

### Statistics

Corrected p values were used for multiple comparisons within and between the groups for normally distributed data. Non-parametric statistical tests were used for nonnormally distributed data. Wilcoxon signed rank test for paired and Wilcoxon rank sum test for comparison in between the two groups. All the analysis was done using R statistical programming language. All data were presented as mean ± SD, p < 0.05 is considered significant.

## Additional Information

**How to cite this article**: Soree, P. *et al*. Raised HIF1α during normoxia in high altitude pulmonary edema susceptible non-mountaineers. *Sci. Rep.* **6**, 26468; doi: 10.1038/srep26468 (2016).

## Figures and Tables

**Table 1:** Baseline anthropometry, duration of high altitude stay and pulmonary functions in HAPE susceptible (HAPE-S) and HAPE resistant (HAPE-R) subjects.

	**HAPE-S (n** = **11)**	**HAPE-R (n** = **11)**	
Age (Yrs)	31.7 ± 3.3	29.8 ± 2.1	NS
Height (cm)	168.3 ± 6	171.2±3.3	NS
Weight (Kg)	70.7 ± 6.3	69.7 ± 7.8	NS
High altitude stay (Yrs)	½–1	2	
FVC (Lt)	4.3 ± 0.4	4.5 ± 0.4	NS
FVC%	96.3 ± 13.7	94 ± 8.3	NS
FEV1 (Lt)	3.5 ± 0.4	3.8 ± 0.3	NS
FEV1/FVC	82.1 ± 4.8	84 ± 4.6	NS
TLC (Lt)	6.2 ± 0.8	6.3 ± 0.6	NS
TLC%	105.7 ± 16.7	100.1 ± 10.1	NS
FRC (Lt)	3.2 ± 0.6	3.4 ± 0.6	NS
FRC%	110.5 ± 20.1	112.3 ± 19.4	NS
DLCO (ml/kg/mm Hg)	33.9 ± 6.1	37.3 ± 6.3	NS
DLCO%	109.3 ± 21	114.4 ± 19.4	NS
VA%	74.8 ± 8.7	75.9 ± 7.3	NS
DLCO/VA	121.6 ± 15.9	124.3 ± 14.6	NS

Values are presented as Mean ± SD. FVC: forced vital capacity; TLC: Total lung capacity; FRC: Functional residual capacity; FEV_1_: Forced expiratory volume in 1 sec; DLCO: pulmonary diffusion capacity for carbon monoxide; VA: alveolar ventilation; NS = Nonsignificant.*p < 0.05 (HAPE-S versus HAPE-R).

**Table 2:** Hemodynamic response to acute hypoxia in: HAPE susceptible (HAPE-S) and HAPE resistant (HAPE-R) subjects.

	**Normoxia**		**Hypoxia**	
	**HAPE-S (n** = **11)**	**HAPE-R (n** = **11)**	**HAPE-S (n** = **11)**	**HAPE-R (n** = **11)**
HR (beats/min)	68 ± 5.9	60.9 ± 6.1^**a**^	83.9 ± 10*	70.4 ± 9.6*^a1^
SBP (mm Hg)	123.1 ± 8.4	120.3 ± 11.9	128.4 ± 11	119.9 ± 8.9
DBP (mm Hg)	71 ± 5.8	67.2 ± 3.8	75.2 ± 6.5	66.4 ± 6.2^**a1**^
Spo_2_ (%)	98.4 ± 1.4	98.5 ± 0.8	66.9 ± 11.7*	78.7 ± 3.8*^a1^
sPpa (mm Hg)	28.2 ± 6.2	22.4 ± 4.6^**a**^	42.2 ± 9.5*	28.4 ± 6.1*^a1^
Ppa (mm Hg)	19.2 ± 3.7	15.7 ± 2.8^**a**^	27.9 ± 6*	19.3 ± 3.7*^a1^

Values are presented as Mean ± SD. HR: heart rate; SBP: systolic blood pressure; DBP: diastolic blood pressure; MAP: mean arterial pressure; Spo_2_: Peripheral oxygen saturation; sPpa: pulmonary artery systolic pressure; Ppa: Pulmonary artery pressure.

^a^p < 0.05 (HAPE-S versus HAPE-R under normoxia); ^a1^p < 0.05 (HAPE-S versus HAPE-R under hypoxia). ^*^normoxia vs hypoxia.

**Table 3:** Venous blood gas, hormonal and biochemical parameters in HAPE susceptible (HAPE-S) and HAPE resistant (HAPE-R) subjects.

	**Group 1 (HAPE-S) (n** = **11)**	**Group 2 (HAPE-R)**
T_3_ (pg/dl)	1.3 ± 0.3	1.0 ± 0.3* (n = 7)
T_4_ (pg/dl)	112.7 ± 37.3	126.4 ± 47.1 (n = 7)
TSH (μIU/ml)	2.0 ± 0.9	2.9 ± 1.8 (n = 7)
pH	7.3 ± 0.04	7.29 ± 0.03 (n = 11)
Pvo_2_ (mm Hg)	24.9 ± 5.4	16.0 ± 4.2* (n = 11)
Svo2%	36.1 ± 13.4	19 ± 7.8* (n = 11)
P50 (mm Hg)	26.3 ± 1.6	24.4 ± 3.2* (n = 11)
ANP (pg/dl)	17.3 ± 9	8.4 ± 7.7* (n = 9)
HIF1α (pg/ml)	320.3 ± 267.5	58.7 ± 33.9* (n = 5)
2–3DPG (nmol/ml)	5.3 ± 1.4	5.2 ± 2.3 (n = 7)

Values are presented as Mean ± SD. T_3_: Triiodothyronine; T_4_: Thyroxine; TSH: Thyroid stimulating hormone; Pvo_2_: partial pressure of venous oxygen; Svo_2_: % venous saturation; ANP: atrial natriuretic peptide; HIF 1α: hypoxia inducible factor 1α; 2–3 DPG: Diphospho glyceric acid.

*p < 0.05 (HAPE-S versus HAPE-R).

**Table 4:** Individual HbA and HbA_2_ fractions of a subgroup of HAPE-S participants.

**HAPE-S Subject No.**	**HbA (area %)**	**HbA**_**2**_ **(area %)**	**Interpretation**
1	69.3	5.4	ß Thalassemia trait
2	60.8	36 (HbE + A_2_)	Hb E heterozygous
3	89.2	2.5	Normal Hb Fraction
4	92.9	3.5	Normal Hb Fraction
